# Transcriptomic Analysis of Cadmium Stressed *Tamarix hispida* Revealed Novel Transcripts and the Importance of Abscisic Acid Network

**DOI:** 10.3389/fpls.2022.843725

**Published:** 2022-04-18

**Authors:** Pei-Long Wang, Xiao-Jin Lei, Yuan-Yuan Wang, Bai-chao Liu, Dan-ni Wang, Zhong-Yuan Liu, Cai-Qiu Gao

**Affiliations:** ^1^State Key Laboratory of Tree Genetics and Breeding, Northeast Forestry University, Harbin, China; ^2^Zhejiang Institute of Subtropical Crops, Zhejiang Academy of Agricultural Sciences, Wenzhou, China

**Keywords:** *Tamarix hispida* Willd, cadmium stress, transcriptomic analysis, differentially expressed genes, H_2_O_2_, ABA

## Abstract

Cadmium (Cd) pollution is widely detected in soil and has been recognized as a major environmental problem. *Tamarix hispida* is a woody halophyte, which can form natural forest on the desert and soil with 0.5 to 1% salt content, making it an ideal plant for the research on response to abiotic stresses. However, no systematic study has investigated the molecular mechanism of Cd tolerance in *T. hispida*. In the study, RNA-seq technique was applied to analyze the transcriptomic changes in *T. hispida* treated with 150 μmol L^–1^ CdCl_2_ for 24, 48, and 72 h compared with control. In total, 72,764 unigenes exhibited similar sequences in the Non-redundant nucleic acid database (NR database), while 36.3% of all these unigenes may be new transcripts. In addition, 6,778, 8,282, and 8,601 DEGs were detected at 24, 48, and 72 h, respectively. Functional annotation analysis indicated that many genes may be involved in Cd stress response, including ion bonding, signal transduction, stress sensing, hormone responses and ROS metabolism. A *ThUGT* gene from the abscisic acid (ABA) signaling pathway can enhance Cd resistance ability of *T. hispida* by regulating the production of ROS under Cd stress and inhibit absorption of Cd. The new transcriptome resources and data that we present in this study for *T. hispida* may facilitate investigation of molecular mechanisms governing Cd resistance.

## Introduction

Heavy metal pollution in soil has become a worldwide problem. It not only inhibits crop growth and reduces yield and quality but also poses a considerable threat to human health (Wu et al., 2007). The heavy metal cadmium is a biologically non-essential element. Cd is highly toxic and migratory with a biological half-life of 10 to 30 years (Suwazono et al., 2009). Therefore, Cd can easily enter the human body through the food chain and accumulate in the body, causing injury to the kidneys, lungs, liver, testicles, brain, bones, and blood system (Hamada et al., 1991).

High concentration of Cd is toxic to plants, but some plants grow under high Cd stress without exhibiting toxic effects. Approximately 10–33% of *Arabidopsis halleri* subsp. *gemmifera* can accumulate more than 100 mg kg^–1^ of Cd in contaminated soil (Bert et al., 2002). Yang et al. (2004) showed no reduction in shoot and root dry matter yields when *S. alfredii* were grown at Cd levels of 200 μmol L^–1^ in nutrient solution. Under natural conditions, the aboveground part of *Noccaea caerulescens* can accumulate up to 164 mg kg^–1^ Cd (Baker et al., 1994). *T. praecox* is a hyperaccumulator plant of zinc, cadmium and lead, and the aboveground part can accumulate up to 5,030 mg kg^–1^ of Cd (Vogel-Mikus et al., 2005). Under Cd pollution level of 25 mg kg^–1^, the Cd content in stems and leaves of *S. nigrum* exceeded 100 mg kg^–1^, and it was greater in shoots than in roots (Wei et al., 2005). These studies suggested that these super enriched plants with good cadmium tolerance can provide the theoretical basis for the study of plant remediation of Cd-contaminated soil.

At present, it is generally believed that accumulation of Cd in plants is primarily reflected in two aspects. On the one hand, at the cellular level, Cd primarily accumulates in the vacuoles and apoplasts of plants. On the other hand, at the organ level, this process is manifested in the epidermal cells, subepithelial cells and epidermal hairs of plants. According to Küpper et al. (2000), mustard mesophyll cells are important sites for Cd accumulation. In addition, Salt and Wagner (1993) reported deposit of large amount of Cd in the leaf epidermis and epidermis hairs in mustard. Recently, researchers found that the vacuolar membrane of rapeseed and *A. thaliana* play an important role in regulating the ion channel protein activity of NO_3_^–^ and Cd, and the vacuolar compartmentalization and cell wall fixation of Cd may be the main physiological reasons for the difference in Cd toxicity resistance between Cd-resistant cultivar Z11 and Cd-sensitive cultivar W10 of rapeseed, which provides a means of synergistically improving the NUE and Cd toxicity of rapeseed (Zhang et al., 2019b). In *A. thaliana*, the defensive protein AtPDF2.5 may chelate cytoplasmic Cd and mediate its efflux, promote Cd accumulation in apoplasts, and regulate plant detoxification and accumulation of Cd (Luo et al., 2019). Therefore, accumulation of Cd in cell walls, vacuoles, epidermal cells or epidermal hair is likely to be one of the ways in which plants achieve detoxification.

In recent years, with of availability of transcriptional data, a growing body of knowledge regarding the genetic basis underlying Cd stress physiological processes has greatly increased our understanding of the molecular mechanism of Cd transcription and toxicity in some Cd hyperaccumulating plants, such as *A. halleri* (Herbette et al., 2006), *Brassica juncea* (Farinati et al., 2010), *S. alfredii* (Gao et al., 2013) and *Noccaea caerulescens* (Halimaa et al., 2014; Milner et al., 2014). At the same time, the molecular mechanisms of Cd stress on some cultivated plants such as cabbage (*Brassica oleracea subsp. capitata f. alba*) (Ba̧czek-Kwinta et al., 2019), pea (*Pisum sativum* L.) (Rodriguez-Serrano et al., 2009), barley (*Hordeum vulgare* L.) (Cao et al., 2014), rice (*Oryza sativa* L.) (Oono et al., 2014), tobacco (*Nicotiana tabacum* L.) (Martin et al., 2012), ramie (*Boehmeria nivea* L.) (Liu et al., 2015) and pakchoi (*Brassica chinensis* L.) (Zhou et al., 2016) were studied.

*Tamarix hispida* is a woody halophyte that grows in arid and semiarid regions. In a previous study, the transcriptome of *T. hispida* treated with NaHCO_3_ was constructed and analyzed to detect the response of *T. hispida* to alkaline treatment (Wang et al., 2013). Some transcription factors, including *ThNAC7*, *ThCRF1*, *ThZFP1*, and *ThbHLH1*, involved in the process of reducing ROS to confer salt or osmotic tolerance in transgenic plant have been cloned (Zang et al., 2015; Ji et al., 2016; Qin et al., 2017; He et al., 2019). There were studies in which multiple *T. hispida* genes enhanced tolerance to Cd. For example, the transfer of the metallothionein gene *ThMT3*, increased resistance to Cd in transgenic tobacco and yeast (Yang et al., 2011; Zhou et al., 2014). Overexpression of vacuolar membrane H^+^-ATPase c subunit gene *ThVHAc1* improved Cd tolerance of *Saccharomyces cerevisiae*, *Arabidopsis*, and *T. hispida*. *ThWRKY7*, a possible upstream gene of *ThVHAc1*, exhibited similar expression patterns as *ThVHAc1* under CdCl_2_ treatment and improved Cd tolerance in *T. hispida* (Gao et al., 2011; Yang et al., 2016).

Investigating transcriptomic response of Cd-stressed leaves would be particularly useful for furthering the genetic improvement of *T. hispida* to Cd stress. To elucidate the initial perception mechanism in response to Cd stimuli in *T. hispida* leaves, we examined gene expression changes at different time points and identified Cd-specific regulatory networks. This study helps to elucidate the mechanism of Cd tolerance in *T. hispida* and provides a useful reference for further exploration in woody plants and used for remediation of heavy metals (Cd) from contamination soils.

## Materials and Methods

### Plant Materials and Cadmium Treatments

Seeds of *T. hispida* sourced from The Turpan Desert Botanical Garden (Xinjiang, 293 China) were germinated in plastic pots containing a mixture of turf peat and sand (1:1 v/v) under constant photoperiod conditions (14/10 h light/dark) with a light intensity of 1,500∼2,000 lx at temperature (24 ± 1°C). After culturing for 3 months in a greenhouse, at least 800 healthy seedlings of similar size (9 cm in height) were selected for Cd treatment. Based on preliminary test results (Gao et al., 2011), 150 μmol L^–1^ CdCl_2_ was used to irrigate the seedlings. At the same time, samples irrigated with fresh water were treated as control. After 24, 48 or 72 h treatment (each treatment contained three separate repeats with at least 200 seedlings), the leaves were washed with clean water and frozen in liquid nitrogen immediately, after which they were stored at −80°C for subsequent experiments. Each sample contains three replicates.

### Determination of Cadmium Concentration and H_2_O_2_-Related Physiological Indices

To detect the Cd concentration of the samples, leaves from samples (each sample contains at least 20 seedlings) containing three replicates for each control, 24, 48 and 72 h were dried at 72°C to a constant weight and then digested with HNO_3_. Subsequently, the Cd ion content was determined using ICP-OES 5110 VDV (Agilent Instruments Inc., CA, United States) (Gdbrijel et al., 2009; Han et al., 2016). The H_2_O_2_ content was detected by a hydrogen peroxide assay kit (Nanjing Jiancheng Bioengineering Institute), and detailed operating procedures were carried out according to the manufacturer‘s instructions. At the same time, hydrogen peroxide (H_2_O_2_) content in the leaves after Cd stress was detected by 3,3-diaminobenzidine (DAB) staining. Briefly, the above mentioned samples were placed in PBS (pH 7.0) solution containing 1 mg mL^–1^ DAB and treated in the dark for 12 h at 37°C. After exposure for 1 h, samples were decolorized with ethanol, and finally, the seedlings were photographed (Daudi and O’Brien, 2012).

### Ribonucleic Acid Extraction, Sequencing and *de novo* Assembly

Total RNA was extracted from the leaf tissues of each sample by CTAB method (Jiang and Zhang, 2003). The degree of degradation of RNA samples was verified by RNase-free agarose gel electrophoresis. The RNA concentration was detected by qubit, and the RNA integrity was accurately detected with an Agilent 2100 Bioanalyzer. Equal quantities of high-quality RNA from the samples was used for the subsequent RNA sequencing.

Complementary DNA libraries were constructed for each of the samples and sequenced on the Illumina HiSeq 2000 platform (Illumina Inc., CA, United States). The original sequenced reads or raw reads containing reads with adapters or low quality were filtered to obtain clean reads. Specifically, reads with adapters were removed, unknown bases (N bases) over 10% and/or low-quality reads (the number of bases with a mass value of Qphred ≤ 20 accounts for more than 50% of the total reads) were removed from each data. Then, the clean reads of the twelve samples with high quality were spliced to construct unique sequences as the reference sequences using the Trinity package (Grabherr et al., 2011). The quality of transcripts were estimated by the value of FPKM (expected number of fragments per kilobase of transcript sequence per million base pairs sequenced).

### Normalization of Gene Expression Levels and Identification of Differentially Expressed Genes

The clean reads of each sample were remapped to reference sequences using RSEM software (Li et al., 2009). RSEM counts the results of the bowtie comparison and further obtains the number of read counts for each sample that was aligned to each gene and performed FPKM conversion to analyze gene expression levels. For genes with more than one alternative transcript, the longest transcript was selected to calculate the FPKM.

To infer transcriptional changes over time under Cd stress conditions, differentially expressed genes (DEGs) after 24, 48, and 72 h of Cd treatment were identified by comparing the expression levels with control. The false discovery rate (FDR) was calculated to adjust the threshold of *p*-value to correct for multiple testing (Rajkumar et al., 2015). Transcripts with a minimal four-fold difference in expression (| log_2_Ratio| ≥2) and an FDR ≤ 0.001 were considered differentially expressed between two time points (Audic and Claverie, 1997). For convenience, DEGs with higher expression levels at 24, 48, and 72 h than control were denoted as ‘‘upregulated,’’ whereas those with the opposite were denoted as ‘‘downregulated.’’ At the same time, Venn diagrams of these differentially expressed genes were made to distinguish the differences between them using online software^1^.

Short Timeseries Expression Miner (STEM) version 1.3.8 was used to analyze expression pattern (Ernst and Bar-Joseph, 2006). To further explore the temporal expression patterns, K-means clustering was applied to the identified DEGs. The DEGs belonging to the same cluster have expression patterns similar to each other. For each genotype, the clustering profiles of DEGs with *p*-values < 0.05 were considered to be significantly different from the reference group.

### Validation of Differentially Expressed Genes With Quantitative Real-Time Polymerase Chain Reaction

Eight genes were randomly selected for quantitative real-time RT-PCR (qRT-PCR) to determine the expression patterns revealed by RNA sequencing. RNA was extracted from *T. hispida* leaves of Cd treated samples (control, 24, 48, and 72 h). The PrimeScript™ RT reagent Kit (Takara) was used for first-strand cDNA synthesis. Primers for qRT-PCR analysis are listed in Supplementary Table 1. β*-actin* (FJ618517) was used as an internal control (Vandesompele et al., 2002). qRT-PCR was performed using a real-time PCR instrument (qTOWER 2.0) (analytik jena, Jena, Germany). The reaction mixture (20 μl) consisted of 10 μl of TransStar*^R^* Top Green qPCR SuperMix (TRANS), 2 μl of cDNA template (equivalent to 500 ng of total RNA), 0.5 μmol L^–1^ of forward and reverse primers. The reaction procedure was as follows: one cycle at 95°C for 3 min, followed by 45 cycles of 95°C for 30 s, 58°C for 15 s, 72°C for 30 s. Three independent replicates were performed to ensure the reproducibility of results. Expression levels of these genes were determined according to the 2^–ΔΔ(Ct)^ (Livak and Schmittgen, 2001). Then, qRT-PCR results and sequencing results were analyzed together to verify the accuracy of the sequencing results.

### Sequence Annotation, Functional Classification, and Biological Pathway Analysis

The unigenes were analyzed for functional annotation and functional classification. After splicing, the unigene sequences were compared with the protein database by Blast, and the annotation included the NCBI Non-redundant nucleic acid database (NR) and the Swiss-port protein sequence database (Swiss-Prot) with a threshold of *e*-value < 0.00001. The GO annotation information was obtained by Blast2GO analysis based on the NR annotation information (Conesa et al., 2005) and classification of all unigenes was performed by WEGO (Ye et al., 2006). At the same time, the Kyoto Encyclopedia of Genes and Genomes (KEGG) was performed to further characterize the metabolic pathways and biological functions of DEGs in the transcriptome.

### Cloning of *ThUGT* Gene in Abscisic Acid Signaling Pathway and It’s Cadmium Resistance Function Analysis

The abscisic acid (ABA) signaling pathway gene *ThUGT* was successfully cloned using F: ATGGCTTCAGAATCCCATGAT and R: TTAGTTAATCCGGCCAC CTTT as primer, then an overexpression vector (*ThUGT*-pROKII) was constructed using F: GCTCTAGAATGGCTTCAGAATCCCATGAT and R: CGGGGTACCTTAG TTAATCCGGCCACCTTT following Wang et al. (2020). Afterward, the overexpression vector strain was transiently transformed into *T. hispida* according to the method of Ji et al. (2014) and treated with 100 μmol L^–1^ CdCl_2_ for 24 h, and the pROKII empty vector transiently transformed seedlings were used as control. Each treatment contained three separate repeats with at least 45 seedlings. Then the expression levels of *ThUGT* in transient overexpression *T. hispida* and control seedlings were analyzed by qRT-PCR. At the same time, the Cd content, ABA content (SenBeiJia Biological Technology Co., Ltd., Nanjing, China) and H_2_O_2_ content (Nanjing Jiancheng Bioengineering Institute) of the samples were determined. DAB, NBT and Evans Blue staining were also performed on each sample (Zhang et al., 2011). Each experiment was repeated three times.

## Results

### Changes of Cadmium and H_2_O_2_ Concentrations in *Tamarix hispida* Subjected to Cadmium Stress

**FIGURE 1:**
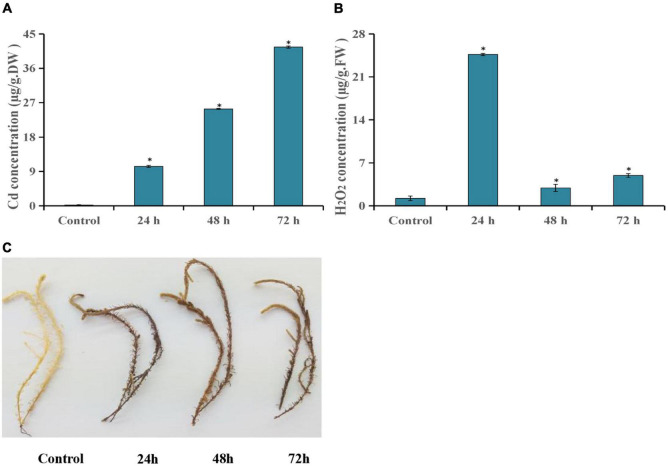
Cd stress analysis of *T. hispida.* The leaves Cd **(A)** and H_2_O_2_ **(B)** concentrations in *T. hispida* at control or under 150 μmol L^– 1^ CdCl_2_ treated for 24, 48, or 72 h. **(C)** DAB staining of *T. hispida* leaves under control or after cadmium stress. * (*P* < 0.05) indicate signification difference compared with control.

In this study, the Cd and H_2_O_2_ contents were detected after *T. hispida* was treated with 150 μmol L^–1^ CdCl_2_. The results showed that Cd concentration showed an upward trend after Cd stress compared with control. Especially at 72 h, the concentration reached a peak with an absolute value of 41.6 mg kg^–1^, which was 227 times that of control (Figure 1A). The H_2_O_2_ concentration was also significantly increased after Cd stress and reached the highest level at 24 h (20.2 times that of control) (Figure 1B). At the same time, the dense DAB staining of the leaves of *T. hispida* was observed at 24 h (Figure 1C).

### Pairwise Comparisons of Transcriptome Between Control and Cadmium Stressed Leaves

**FIGURE 2:**
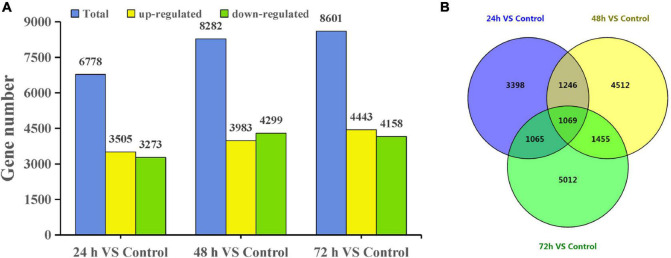
DEGs of *T. hispida* under cadmium treatments. **(A)** Gene number analysis of DEGs between the cadmium treated transcriptomes compared with the control. **(B)** Venn diagrams of these DEGs.

Using a cutoff of four-fold difference in gene expression, 3,505, 3,983, and 4,443 upregulated genes and 3,273, 4,299, and 4,158 downregulated genes were identified at 24, 48, and 72 h, respectively, compared with those in the control (Figure 2A). Among these genes, 2,673, 3,058, and 3,077 DEGs at 24, 48, and 72 h had no similar sequences in the NR database, respectively (Supplementary Table 2). Interestingly, the expression of many DEGs changed significantly only at a certain time point. For example, there were 5,012 DEGs at 72 h and there were 1,069 DEGs common to all three treatment time points compared with the control (Figure 2B).

**FIGURE 3:**
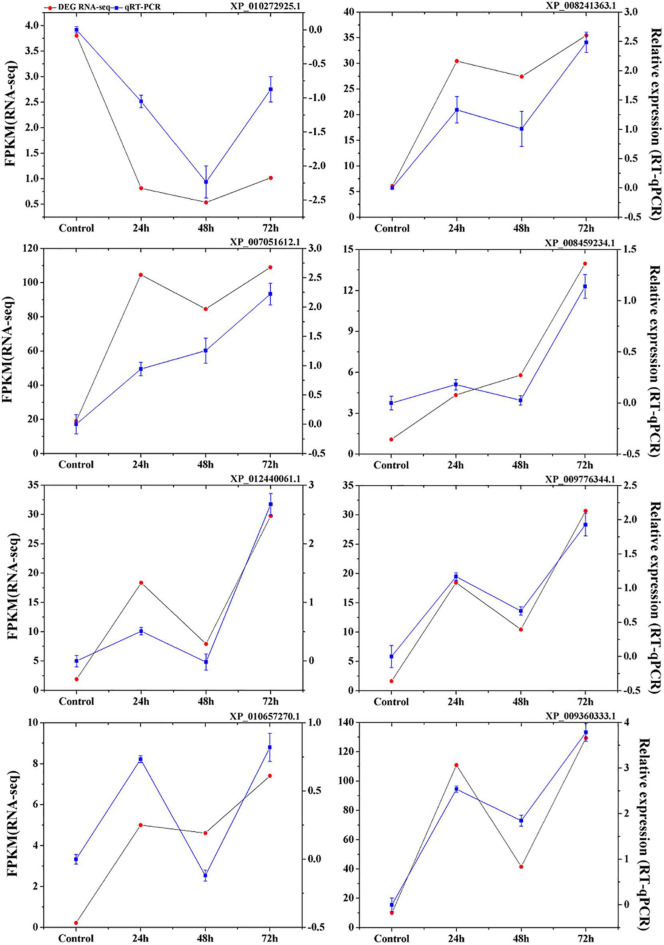
Verification of eight selected DEGs by qRT-PCR. Comparison of RNA-seq data (Red point) with qRT-PCR data (Blue point). The normalized expression level (FPKM; expected number of Fragments Per Kilobase of transcript sequence per Millions base pairs sequenced) of RNA-seq is indicated on the *y*-axis to the left. The relative qRT-PCR expression level of selected DEGs is shown on the *y*-axis to the right. β*-actin* was used as the internal control. Three biological replicates were used.

To validate the expression data obtained from RNA sequencing, eight genes were randomly selected from the identified DEGs to perform qRT-PCR analysis. The results showed a strong correlation between the RNA sequencing and qRT-PCR data (Figure 3), which supports the reliability of the expression results generated by RNA sequencing.

**FIGURE 4:**
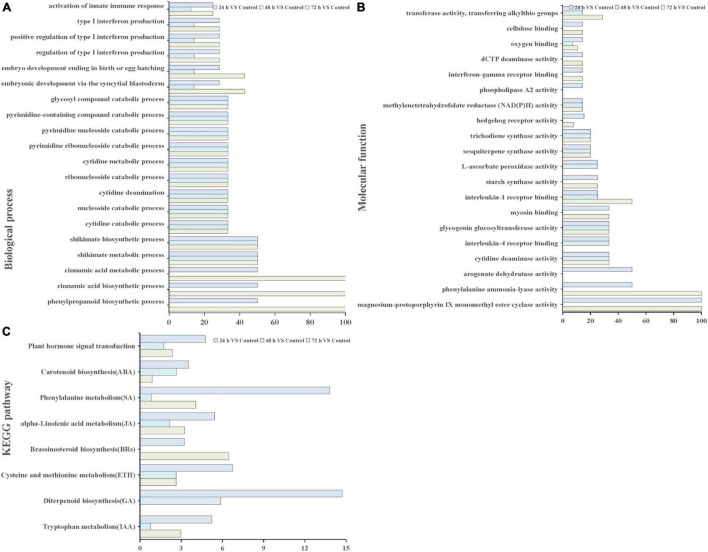
GO terms and KEGG pathways involved in ROS and hormones production analysis. GO analysis of biological process terms **(A)**, molecular function terms **(B,C)** KEGG pathways involved in hormones between the cadmium treated transcriptomes compared with the control. The *x*-axis in **(A,B)** indicates the percentage of DEG snumbers vs. background gene numbers in each GO term. The *x*-axis in **(C)** indicates the percentage of DEGs numbers vs. background gene numbers in each KEGG pathway.

Through GO and KEGG pathway enrichment analysis, the function of the DEGs were characterized. GO annotation suggested that biological processes and molecular functions related to ROS functions and biosynthetic and metabolic processes were enriched among the DEGs at different time points (Figures 4A,B). In biological processes, biosynthetic, and metabolic processes, DEGs were enriched at all three time points (Figure 4A). In addition, the molecular function term “phenylalanine ammonia-lyase activity” was enriched at 24 and 72 h. The term “methylenetetrahydrofolate reductase NAD(P)H activity” occurred at all three time points (Figure 4B). These results indicated that these genes or proteins participate in hormone and ROS metabolism play crucial roles in the *T. hispida* response to Cd stress.

The Kyoto Encyclopedia of Genes and Genomes pathway enrichment analysis results showed that 90, 91, and 105 pathways were categorized from the pairwise comparisons between 24 h vs. control, 48 h vs. control, and 72 h vs. control, respectively. Based on these results, the DEGs involved in the biosynthetic pathways of seven hormones and one pathway for “plant hormone signal transduction” were enriched (Figure 4C). At the same time, we observed that the number of genes involved in six hormone synthetic pathways were the highest at 72 h, whereas brassinosteroid biosynthesis pathways peaked at 24 h. Interestingly, six hormone synthetic pathways were included in the top 30 pathways from 24 h vs. control (Table 1). In the top 20 pathways in 24 h vs. control, five hormone synthesis pathways were found. These results demonstrated that the expression of genes involved in hormone synthesis may play an important role in *T. hispida* response to Cd stress.

**TABLE 1:** Top 30 KEGG pathways based on the percentage of DEGs in 24 h vs. control.

Term	Total	24 h vs. control		48 h vs. control		72 h vs. control	
		DEGs	%	DEGs	%	DEGs	%
Glucosinolate biosynthesis	7	2	28.6	1	14.3	2	28.6
Photosynthesis – antenna proteins	48	10	20.8	1	2.1	10	20.8
Cutin, suberin, and wax biosynthesis	77	7	9.1	2	2.6	9	11.7
Brassinosteroid biosynthesis (BRs)	31	2	6.5	0	0.0	1	3.2
Flavonoid biosynthesis	73	4	5.5	1	1.4	11	15.1
Phenylpropanoid biosynthesis	372	19	5.1	12	3.2	49	13.2
Stilbenoid, diarylheptanoid, and gingerol biosynthesis	61	3	4.9	0	0.0	10	16.4
Cyanoamino acid metabolism	161	7	4.4	6	3.7	13	8.1
Phenylalanine metabolism (SA)	123	5	4.1	1	0.8	17	13.8
Nitrogen metabolism	104	4	3.9	4	3.9	11	10.6
Arachidonic acid metabolism	57	2	3.5	1	1.8	5	8.8
Phenylalanine, tyrosine, and tryptophan biosynthesis	206	7	3.4	8	3.9	9	4.4
alpha-Linolenic acid metabolism (JA)	184	6	3.3	4	2.2	10	5.4
Tryptophan metabolism (IAA)	134	4	3.0	1	0.8	7	5.2
Alanine, aspartate and glutamate metabolism	241	7	2.9	4	1.7	22	9.1
Ascorbate and aldarate metabolism	214	6	2.8	7	3.3	19	8.9
Pentose phosphate pathway	215	6	2.8	7	3.3	15	7.0
Amino sugar and nucleotide sugar metabolism	408	11	2.7	6	1.5	24	5.9
Cysteine and methionine metabolism (ETH)	342	9	2.6	9	2.6	23	6.7
Terpenoid backbone biosynthesis	190	5	2.6	3	1.6	4	2.1
Taurine and hypotaurine metabolism	76	2	2.6	0	0.0	7	9.2
Steroid biosynthesis	123	3	2.4	1	0.8	8	6.5
Plant hormone signal transduction	633	15	2.4	11	1.7	30	4.7
Butanoate metabolism	128	3	2.3	1	0.8	2	1.6
Photosynthesis	131	3	2.3	3	2.3	12	9.2
Carbon fixation in photosynthetic organisms	306	7	2.3	8	2.6	28	9.2
Plant-pathogen interaction	482	11	2.3	13	2.7	20	4.2
Glutathione metabolism	264	6	2.3	8	3.0	18	6.8
Pentose and glucuronate interconversions	221	5	2.3	4	1.8	13	5.9
Sphingolipid metabolism	133	3	2.3	4	3.0	5	3.8

*Hormone-related KEGG pathways have a gray background.*

### Analysis of Temporal Expression Pattern of Genes

**FIGURE 5:**
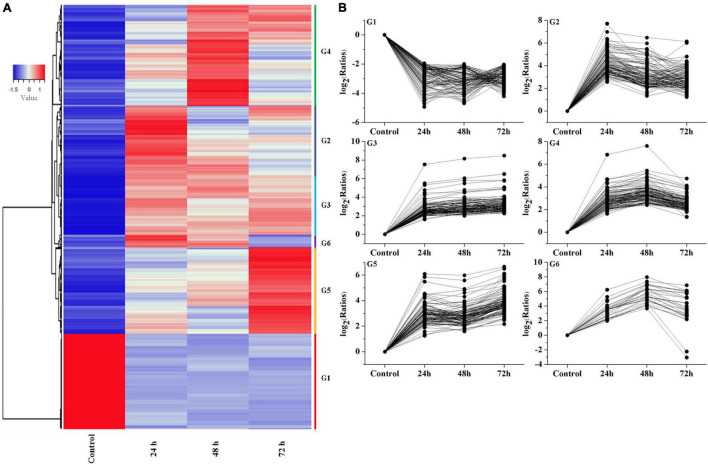
Analysis of temporal expression pattern of genes. **(A)** Heat map analysis of DEGs. **(B)** Expressive trend pattern analysis of DEGs. The value of ratios take the base 2 logarithm were used to analysis each gene‘s expression trend in every group. For each gene, ratios = FPKM of the gene in sample/FPKM value of the gene in control.

Hierarchical clustering produced six groups with similar expression trends to those of K-means clustering. Specifically, 1,069 qualifying genes were categorized into six groups (referred to as G1, G2, G3, G4, G5, and G6), comprising 240, 110, 384, 142, 164, and 29 genes, respectively (Figure 5), of which the G2, G3, G4, G5, and G6 groups mainly showed an upregulated trend under Cd treatment.

The expression of G1 group showed a downregulation trend, and reached its lowest value at 48 h, then slightly increased at 72 h. The G2 group mainly reached a peak at 24 h, then showed a downward trend and slightly increased at 72 h. The G3 group showed an upregulation trend at the beginning (24 h) followed by stable expression in the subsequent stage. The G4 group showed a continuous increasing trend at 24 h and 48 h. Interestingly, the G5 group showed an increasing expression trend during the whole stage and peaked at 72 h. The G6 group showed an upregulation trend at 24 and 48 h and a slight decrease at 72 h. From these results, we can assume that the genes in G3 and G5 group can be rapidly induced by Cd stress, and the genes in G2, G4, and G6 group showed a time-dependent trend in the process. In contrast, the genes in G1 group showed inhibition trend in response to cadmium stress.

In the GO functional annotation analysis, the number of genes in each group based on their biological pathway, molecular function and cellular component were counted. The results showed that all selected DEGs were involved in 282 biological pathways. Among them, the number of DEGs involved in protein binding, ATP binding, and DNA binding were 88, 52, and 45, respectively (Table 2).

**TABLE 2:** The top 20 GO molecular function based on number of common DEGs.

Gene Ontology Molecular Function	DEG_item
protein binding	88
ATP binding	52
DNA binding	45
zinc ion binding	33
nucleic acid binding	31
oxidoreductase activity	28
protein kinase activity	24
metal ion binding	21
structural constituent of ribosome	20
RNA binding	18
transcription factor activity, sequence-specific DNA binding	17
calcium ion binding	14
transmembrane transporter activity	14
catalytic activity	13
hydrolase activity, hydrolyzing O-glycosyl compounds	12
GTP binding	10
electron carrier activity	9
GTPase activity	9
heme binding	9
nucleotide binding	8

In total, the K-means/hierarchical clustering and GO function annotation results indicated that the genes related to hormones were significantly affected during Cd stress in *T. hispida* leaves. Hence, the DEGs involved in the metabolism of hormones and their signaling pathways were further explored systematically.

### Differentially Expressed Genes Involved in Hormone Biosynthetic Pathways

To further explore the genes involved in hormone biochemical pathways following Cd stress treatment, the DEGs involved in the seven hormone biosynthesis or metabolism KEGG pathways (ABA, ETH, IAA, SA, GA, BRs, and JA) were analyzed (Table 1). The number of genes in the ABA, SA, ETH, IAA, GA, and JA biosynthetic or metabolism pathways primarily increased, while BR biosynthesis showed decreasing trends (Figure 4C).

Under Cd stress, 7 DEGs were identified in the ABA-related pathway, including ABA β-glucosyltransferase, zeta-carotene desaturase (PLN02487), isoprenoid biosynthesis enzymes (IBe), carotene beta-ring hydroxylase (PLN02738), antheraxanthin epoxidase/zeaxanthin epoxidase (PLN02927) and cytochrome P450. Three of them were induced, especially *ThUGT* (ABA β-glucosyltransferase), which appeared at 24 and 72 h with 3.7 and 4.2-fold increased, respectively. In contrast, the expression of PLN02487, PLN02738, PLN02927, and cytochrome P450 showed reduced expression trend.

Thirty DEGs in ethylene biosynthetic pathway were detected. Among the metabolic processes, there were nine beta-eliminating lyase genes, four hypothetical proteins, two 1-aminocyclopropane-1-carboxylate oxidases and two 5-methyltetrahydropterin glutamate homocysteine methyltransferases. The expression of these 17 genes were upregulated. In contrast, the expression of genes involved in homocysteine S-methyltransferase, 1-aminocyclopropane-1-carboxylate synthase, ARD/ARD’ family and S-adenosyl-methionine synthase were downregulated.

Hormones content, antioxidant activities, and downstream signals are induced in response to environmental stresses to control cell stability and mitigate the negative effects of ROS (Abdullah et al., 2021; Heidari et al., 2021). These results indicated that the key regulatory components of the biosynthetic pathways in ABA and ethylene changed significantly during the *T. hispida* response to Cd stress. In addition, there are also many genes involved in BRs, JA, GA, IAA, and SA, also responded to Cd stress.

### *ThUGT*-Overexpressing *Tamarix hispida* Increased Abscisic Acid Content and Cadmium Tolerance

**FIGURE 6:**
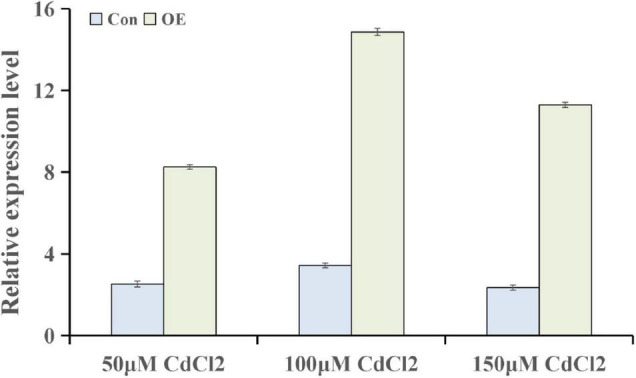
The expression levels of *ThUGT* gene in transiently transformed *T. hispida* under different CdCl_2_ stress conditions.

**FIGURE 7:**
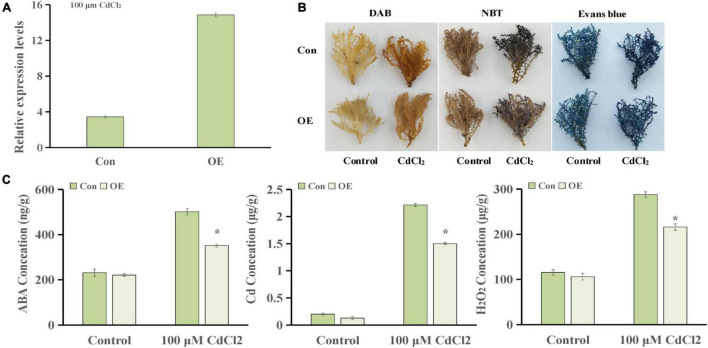
Cadmium tolerance analysis of *ThUGT* gene. **(A)** qRT-PCR analysis **(B)** staining analysis and **(C)** physiological indicators determination of *ThUGT* transgenic *T. hispida* under 100 μmol L^– 1^ CdCl_2_ stress. * (*P* < 0.05) indicate signification difference compared with control.

To further explore the genes involved in hormone pathways following Cd stress treatment, the ABA-related KEGG pathway after each stress treatment point was analyzed. The results showed that ABA pathway-related genes mainly participated in phytoene, lycopene, zeaxanthin, abscisate and lutein biosynthetic processes (Supplementary Figure 1). *ThUGT*, a predicted ABA β-glucosyltransferase gene, was one of the DEGs in the ABA signaling pathway, and the expression level was induced (3.7 and 4.2 times of the control at 24 and 72 h, respectively). Therefore, *ThUGT* was transiently transformed into *T. hispida*. Pre-experimental results showed that in case of treatment with 100 μmol L^–1^ CdCl_2_ after 24 h, the relative expression level of *ThUGT* gene had the largest difference with the normal conditions (Figure 6). The results of qRT-PCR showed that the expression level of *ThUGT* was significantly higher in the overexpressing plants than in the control plants (Figure 7A).

The staining analysis showed that the overexpressing plants were stained lighter after CdCl_2_ stress treatment compared with the control plants (Figure 7B). The results obtained with physiological indicators showed that the ABA content in *ThUGT-*overexpressing *T. hispida* and the control plants was effectively increased under 100 μmol L^–1^ CdCl_2_ treatment, while in the *ThUGT* transgenic plants, it increased less than in the control (Figure 7C). At the same time, the Cd ion content in both the overexpression plants and the control plants increased significantly after Cd treatment, but the Cd ion content in the overexpressing plants was significantly less than that of the control (Figure 7C). The H_2_O_2_ content in overexpression plants was lower than that of the control after Cd treatment (Figure 7C). These results suggested that the *ThUGT* transgenic plants eliminated more ROS and inhibited the absorption of Cd to a certain extent by *T. hispida* under cadmium stress, thereby enhancing the cadmium resistance of *T. hispida*.

## Discussion

When plants are under heavy metal stress, it can produce a series of responses to relieve the toxic effects of heavy metals. Some reports suggested that the production of glutathione, phytochelatin, and metal chelates or chaperones, which can bind to heavy metal ions and then transport them out of cells. In addition, antioxidant enzymes, such as superoxide dismutase (SOD), peroxidase (POD), glutathione reductase (GR), ascorbate peroxidase (APX), and glutathione peroxidase (GPX), were produced to scavenge oxygen free radicals generated by heavy metals (Emamverdian et al., 2015).

In this work, we used RNA-seq to explore the time course of the response mechanism in *T. hispida* under Cd stress. Our study showed that 114,292 unigenes were obtained after the transcriptome data were spliced and 36.3% of the unigenes were novel transcripts under Cd stress. Further analysis found that 6,778 DEGs were detected at 24 h and then increased to 8,282 and 8,601 at 48 and 72 h, respectively (Figure 2A), which indicated that an increasing number of DEGs were induced or activated under Cd stress in *T. hispida*.

H_2_O_2_ is a product of aerobic metabolism of cells, and its production is increased under various stresses. It not only has the effect of damaging biological macromolecules and thereby harming cells, it is also an important signal molecule that induce the expression of a series of defense genes in the cell, improve the activity of protective enzymes to remove active oxygen, prevent its excessive accumulation under adversity conditions, and protect plants from damage (Moller, 2001; Mittler, 2002). Interestingly, Cd content increased with increasing treatment time (Figure 1A), while the H_2_O_2_ content peaked at 24 h (Figures 1B,C). At the same time, we also found that many studies reported H_2_O_2_ functions a secondary messenger during plant development and defense to abiotic stress (Avshalumov et al., 2003; Jiang et al., 2012; Khalili et al., 2014; Liu et al., 2016; Saxena et al., 2016). Therefore, we suggest that H_2_O_2_ may be serving as a secondary messenger to induce the expression of stress-related proteins in the early stage of cadmium stress and initiate the development of systemic acquired resistance in *T. hispida*. This plant synthesizes many ROS-clearing genes, thereby effectively eliminating excess H_2_O_2_ to help *T. hispida* tolerate Cd stress. Consistent with this property, the results of the DEGs at 24 h showed that 1/6 DEGs participated in the redox reaction, the transport of ions, and the synthesis of signal substances (Supplementary Table 3), which have important functions in plant stress resistance.

In previous studies, multiple transcription factors were reported to be involved in the scavenging of ROS or increasing SOD and POD activities in *T. hispida* to improve the salt tolerance or osmotic stress ability of plants (Zang et al., 2015; Ji et al., 2016; Qin et al., 2017; He et al., 2019). Many studies on transcriptome analyses of Cd-treated plants have found that it is mainly related to the pathways of “ROS-scavenging enzymes” (Guo et al., 2017; Gupta et al., 2017), “cell wall alternation and strengthening” (Fan et al., 2011; Wan and Zhang, 2012; Xu et al., 2015), “lipid oxidation” (Guo et al., 2017), “auxin biosynthesis and metabolism” (Yue et al., 2016), and “nitric oxide-mediated homeostasis” (Zuccarelli et al., 2017).

In *T. hispida*, the GO analysis showed in the biological pathway that “oxidation–reduction process” was the top enriched term, and “oxidoreductase activity” in the molecular function also ranked in the top 10 (Figures 4A,B). These results indicated that the expression of antioxidant and redox homeostasis-related genes play important role in response to Cd stress in *T. hispida*.

Abscisic acid, a widely known phytohormone involved in the plant response to abiotic stress, plays a vital role in mitigating Cd^2+^ toxicity in herbaceous species. Studies have found that when plants were exposed to Cd, endogenous ABA levels were increased in plant cells (Sharma and Kumar, 2002). Several other studies demonstrated that the application of ABA can reduce Cd accumulation in crops (Hsu and Kao, 2003, 2005; Uraguchi et al., 2009; Fan et al., 2014). Fan et al. (2014) reported that ABA treatment correlates with the downregulation of ABA-inhibited *IRON-REGULATED TRANSPORTER 1* (*IRT1*) to decrease Cd accumulation. Through interaction with MYB49, the ABI5 represses MYB49 binding to the downstream genes *bHLH38*, *bHLH101*, *HIPP22*, and *HIPP44*, which result in the inactivation of *IRT1* and reduced Cd uptake (Zhang et al., 2019a).

The results of KEGG pathway analysis showed that seven main phytohormones participate in Cd stress in *T. hispida.* Among the phytohormones, the input numbers vs. background gene numbers for ABA pathways increased over time (Figure 4C), with ratios of 0.9, 2.7, and 3.5% being observed at 24, 48, and 72 h, respectively. By GO function analysis, we found that seven DEGs were involved in the ABA signaling pathway, three of which were upregulated (Supplementary Figure 1). In particular, the expression of *ThUGT* gene was clearly induced. The ABA content in transient overexpression *ThUGT T. hispida* plants was significantly increased after cadmium stress treatment, but it was less compared with the control (Figure 7C). Moreover, increase in the H_2_O_2_ content and Cd content was less in OE plants than the control after Cd stress (Figure 7C), which was consistent with the results reported in previous studies, indicating that the *ThUGT* gene has the ability to remove ROS under Cd stress and enhance the tolerance of *T. hispida* to Cd stress.

Uridine diphosphate-glucosyltransferases (UGTs) are a family of proteins involved in physiological responses to the inactivation of many glycosylation hormones (Kleczkowski et al., 1995). The overexpression of *UGT74E2* gene in *A. thaliana* can improve the resistance to drought and salt stress by regulating the ABA dynamic balance (Tognetti et al., 2010). ABA β-glucosyltransferase belongs to the UGT family and is a key enzyme in the ABA catabolism binding pathway. This enzyme plays an important role in maintaining the normal physiological level of ABA. The ABA β-glucosyltransferase gene *AtUGT71B6* in *A. thaliana* regulates intracellular ABA balance (Priest et al., 2006; Dong et al., 2014). The *Phaseolus vulgaris PvABAGT* gene regulates the ABA balance and stress response in adversity during bean development (Xu et al., 2002).

In *Beta vulgaris*, the ROS produced by a plasma membrane NADPH oxidase may act as a signal to induce *BvGT* (UGT family gene) expression after wounding and bacterial infiltration (Sepulveda-Jimenez et al., 2004). Oxidative stress and conditions that promote cell death could induce the expression of glucosyltransferase genes and produce transportable glucosides that function as ROS scavengers (Mazel and Levine, 2002). Therefore, we concluded that *ThUGT* may play a crucial role in the ABA conjugation pathway and in adaptation to Cd stress by inducing ROS scavengers to deduce H_2_O_2_.

## Conclusion

The present study identified novel transcripts, gene structures, and DEGs in *T. hispida* under Cd stress. In total, 114,292 unigenes were identified. The transcripts identified in this study will serve as a valuable genomic resource for future studies. Among these genes, a large number of genes were related to ROS clearance and hormone signals, which may facilitate the analysis of ABA signaling pathways. The overexpression of related a gene *ThUGT* reduced the accumulation of Cd in *T. hispida* under high CdCl_2_ stress. These results will help to establish a foundation for future research to improve cadmium tolerance in *T. hispida* and other plants.

## RNA-Seq Data Submitted to Public Database

The data presented in the study are deposited in the NCBI repository, accession number PRJNA795701 (https://www.ncbi.nlm.nih.gov/bioproject/PRJNA795701).

## Data Availability Statement

The raw data supporting the conclusions of this article will be made available by the authors, without undue reservation.

## Author Contributions

P-LW and C-QG conceived and designed the experiments and wrote the manuscript. P-LW and Y-YW performed the experiments. P-LW and X-JL analyzed the data. All authors provided editorial advice.

## Conflict of Interest

The authors declare that the research was conducted in the absence of any commercial or financial relationships that could be construed as a potential conflict of interest.

## Publisher’s Note

All claims expressed in this article are solely those of the authors and do not necessarily represent those of their affiliated organizations, or those of the publisher, the editors and the reviewers. Any product that may be evaluated in this article, or claim that may be made by its manufacturer, is not guaranteed or endorsed by the publisher.

## Footnotes

1
http://bioinfogp.cnb.csic.es/tools/venny/index2.0.2.html

## Supplementary Material

Supplementary Figure 1 Signaling pathways involved in ABA. The genes associated with ABA were marked in different colors. Red: up-regulated genes. Blue: down-regulated genes.

## Abbreviations

ABA: abscisic acid
APX: ascorbate peroxidase
Cd: cadmium
cDNA: complementary DNA
DAB: 3,3-diaminobenzidine
DEGs: differentially expressed genes
ETH: Ethylene
FDR: false discovery rate
FPKM: expected number of fragments per kilobase of transcript sequence per million base pairs sequenced
GA: gibberellin
GO: Gene Ontology
GPX: glutathione peroxidase
GR: glutathione reductase
H_2_O_2_: hydrogen peroxide
IAA: indole-3-acetic acid
JA: jasmonic acid
KEGG: the Kyoto Encyclopedia of Genes and Genomes
N bases: unknown bases
NBT: Nitro blue tetrazolium
NR: Non-redundant nucleic acid database
POD: peroxidase
qRT-PCR: quantitative real-time polymerase chain reaction
RNA: ribonucleic acid
ROS: reactive oxygen species
SA: Salicylic acid
SOD: superoxide dismutase
STEM: Short Time-series Expression Miner
Swiss-Prot: Swiss-port protein sequence database
*T. hispida*: *Tamarix hispida* Willd
UGTs: uridine diphosphate glucosyltransferases.

## References

[B1] Abdullah, Faraji S. Mehmood F. Malik H. M. T. Ahmed I. Heidari P. (2021). The GASA gene family in cacao (*Theobroma cacao*, Malvaceae): genome wide identification and expression analysis. *Agronomy* 11 1–20.

[B2] Audic S. Claverie J. M. (1997). The significance of digital gene expression profiles. *Genome Res.* 7 986–995. 10.1101/gr.7.10.986 9331369

[B3] Avshalumov M. V. Chen B. T. Marshall S. P. Pena D. M. Rice M. E. (2003). Glutamate-dependent inhibition of dopamine release in striatum is mediated by a new diffusible messenger, H_2_O_2_. *J. Neurosci.* 23 2744–2750. 10.1523/JNEUROSCI.23-07-02744.2003 12684460 PMC6742066

[B4] Ba̧czek-Kwinta R. Juzoń K. Borek M. (2019). Photosynthetic response of cabbage in cadmium-spiked soil. *Photosynthetica* 57 731–739.

[B5] Baker B. J. M. Reeves R. D. HAjar A. S. M. (1994). Heavy metal accumulation and tolerance in British populations of the metallophyte *Thlaspi caerulescens* J. & C. Presl (Brassicaceae). *New Phytol.* 127 6l–68. 10.1111/j.1469-8137.1994.tb04259.x 33874394

[B6] Bert V. Bonnin I. Saumitou-Laprade P. Patrick de Laguérie P.d Daniel Petit D. (2002). Do arabidopsis halleri from nonmetallicolous populations accumulate zinc and cadmium more effectively than those from metallicolous populations. *New Phytol.* 155 47–57. 10.1046/j.1469-8137.2002.00432.x 33873296

[B7] Cao F. B. Chen F. Sun H. Y. Zhang G. P. Chen Z. H. Wu F. B. (2014). Genome-wide transcriptome and functional analysis of two contrasting genotypes reveals key genes for cadmium tolerance in barley. *BMC Genomics* 15:611. 10.1186/1471-2164-15-611 25038590 PMC4117959

[B8] Conesa A. Götz S. García-Goìmez J. M. Terol J. Taloìn M. Robles M. (2005). Blast2GO, a universal tool for annotation, visualization and analysis in functional genomics research. *Bioinformatics* 21 3674–3676. 10.1093/bioinformatics/bti610 16081474

[B9] Daudi A. O’Brien J. S. (2012). Detection of hydrogen peroxide by DAB staining in *Arabidopsis*. *Bio Protoc.* 2:e263. 27390754 PMC4932902

[B10] Dong T. Xu Z. Y. Park Y. Kim D. H. Lee Y. Hwang I. (2014). Abscisic acid uridine diphosphate glucosyltransferases play a crucial role in abscisic acid homeostasis in *Arabidopsis*. *Plant Physiol.* 165 277–289. 10.1104/pp.114.239210 24676855 PMC4012586

[B11] Emamverdian A. Ding Y. L. Mokhberdoran F. Xie Y. F. (2015). Heavy metal stress and some mechanisms of plant defense response. *Sci. World J.* 2015:756120. 10.1155/2015/756120 25688377 PMC4321847

[B12] Ernst J. Bar-Joseph Z. (2006). STEM, a tool for the analysis of short time series gene expression data. *BMC Bioinformatics* 7:191. 10.1186/1471-2105-7-191 16597342 PMC1456994

[B13] Fan J. L. Wei X. Z. Wan L. C. Zhang L. Y. Zhao X. Q. Liu W. Z. (2011). Disarrangement of actin filaments and Ca^2+^ gradient by CdCl2 alters cell wall construction in *Arabidopsis thaliana* root hairs by inhibiting vesicular trafficking. *J Plant Physiol.* 168 1157–1167. 21497412 10.1016/j.jplph.2011.01.031

[B14] Fan S. K. Fang X. Z. Guan M. Y. Ye Y. Q. Lin X. Y. Du S. T. (2014). Exogenous abscisic acid application decreases cadmium accumulation in *Arabidopsis* plants, which is associated with the inhibition of IRT1-mediated cadmium uptake. *Front. Plant Sci.* 5:721. 10.3389/fpls.2014.00721 25566293 PMC4267193

[B15] Farinati S. DalCorso G. Varotto S. Furini A. (2010). The *Brassica juncea* BjCdR15, an ortholog of *Arabidopsis* TGA3, is a regulator of cadmium uptake, transport and accumulation in shoots and confers cadmium tolerance in transgenic plants. *New Phytol.* 185 964–978. 10.1111/j.1469-8137.2009.03132.x 20028476

[B16] Gao C. Q. Wang Y. C. Jiang B. Liu G. F. Yu L. L. Wei Z. G. (2011). A novel vacuolar membrane H^+^-ATPase c subunit gene (ThVHAc1) from *Tamarix hispida* confers tolerance to severalabiotic stresses in *Saccharomyces cerevisiae*. *Mol. Biol. Rep.* 38 957–963. 10.1007/s11033-010-0189-9 20526814

[B17] Gao J. Sun L. Yang X. E. Liu J. X. (2013). Transcriptomic analysis of cadmium stress response in the heavy metal hyperaccumulator *Sedum alfredii* hance. *PLoS One* 8:e64643. 10.1371/journal.pone.0064643 23755133 PMC3670878

[B18] Gdbrijel O. Davor R. Zed R. Marija R. Monika Z. (2009). Cadmium accumulation by muskmelon under salt stress in contaminated organic soil. *Sci. Total Environ.* 407 2175–2182. 10.1016/j.scitotenv.2008.12.032 19162301

[B19] Grabherr M. G. Haas B. J. Yassour M. Levin J. Z. Thompson D. A. Amit I. (2011). Full-length transcriptome assembly from RNA-Seq data without a reference genome. *Nat. Biotechnol.* 29 644–652. 10.1038/nbt.1883 21572440 PMC3571712

[B20] Guo Q. Meng L. Zhang Y. N. Mao P. C. Tian X. X. Li S. S. (2017). Antioxidative systems, metal ion homeostasis and cadmium distribution in *Iris lactea* exposed to cadmium stress. *Ecotoxicol. Environ. Saf.* 139 50–55. 10.1016/j.ecoenv.2016.12.013 28110045

[B21] Gupta D. K. Pena L. B. Romero-Puertas M. C. Hernández A. Inouhe M. Sandalio L. M. (2017). NADPH oxidases differentially regulate ROS metabolism and nutrient uptake under cadmium toxicity. *Plant Cell Environ.* 40 509–526. 10.1111/pce.12711 26765289

[B22] Halimaa P. Lin Y. F. Ahonen V. H. Blande D. Clemens S. Gyenesei A. (2014). Gene expression differences between *Noccaea caerulescens* ecotypes help identifying candidate genes for metal phytoremediation. *Environ. Sci. Technol.* 48 3344–3353. 10.1021/es4042995 24559272

[B23] Han Y. Wang S. Zhao N. Deng S. Zhao C. Li N. (2016). Exogenous abscisic acid alleviates cadmium toxicity by restricting Cd^2+^ influx in *Populus euphratica* cells. *J. Plant Growth Regul.* 35 827–837.

[B24] Hamada T. Nakano S. Iwai S. Tanimoto A. Ariyoshi K. Koide O. (1991). Pathological study on beagles after long-term oral administration of cadmium. *Toxicol. Pathol.* 19 138–147. 10.1177/019262339101900208 1771367

[B25] He Z. Li Z. Lu H. Huo L. Wang Z. Wang Y. (2019). The NAC protein from *Tamarix hispida*, ThNAC7, confers salt and osmotic stress tolerance by increasing reactive oxygen species scavenging capability. *Plants (Basel)* 8:221. 10.3390/plants8070221 31336966 PMC6681344

[B26] Herbette S. Taconnat L. Hugouvieux V. Piette L. Magniette M. L. M. Cuine S. (2006). Genome-wide transcriptome profiling of the early cadmium response of *Arabidopsis* roots and shoots. *Biochimie* 88 1751–1765. 10.1016/j.biochi.2006.04.018 16797112

[B27] Heidari P. Amerian M. R. Barcaccia G. (2021). Hormone profiles and antioxidant activity of cultivated and wild tomato seedlings under low-temperature stress. *Agronomy* 11 1–16.

[B28] Hsu Y. T. Kao C. H. (2003). Role of abscisic acid in cadmium tolerance of rice (*Oryza sativa* L.) seedlings. *Plant Cell Environ.* 26 867–874. 10.1046/j.1365-3040.2003.01018.x 12803614

[B29] Hsu Y. T. Kao C. H. (2005). Abscisic acid accumulation and cadmium tolerance in rice seedlings. *Physiol. Plant.* 124 71–80. 10.1111/j.1399-3054.2005.00490.x

[B30] Ji X. Nie X. Liu Y. Zheng L. Zhao H. Zhang B. (2016). A bHLH gene from *Tamarix hispida* improves abiotic stress tolerance by enhancing osmotic potential and decreasing reactive oxygen species accumulation. *Tree Physiol.* 36 193–207. 10.1093/treephys/tpv139 26786541

[B31] Ji X. Y. Zheng L. Liu Y. J. Nie X. G. Liu S. N. Wang Y. C. (2014). A transient transformation system for the functional characterization of genes involved in stress response. *Plant Mol. Biol. Rep.* 32 732–739. 10.1007/s11105-013-0683-z

[B32] Jiang J. X. Zhang T. Z. (2003). Extraction of total RNA in cotton tissues with ctab-acidic phenolic method. *Acta Gossypii Sin.* 15 166–167.

[B33] Jiang Y. P. Cheng F. Zhou Y. H. Xia X. J. Mao W. H. Shi K. (2012). Hydrogen peroxide functions as a secondary messenger for brassinosteroids-induced CO_2_ assimilation and carbohydrate metabolism in *Cucumis sativus*. *J. Zhejiang Univ. Sci. B* 13 811–823. 10.1631/jzus.B1200130 23024048 PMC3468824

[B34] Khalili M. Hasanloo T. Safdari Y. (2014). Hydrogen peroxide acts as a secondary messenger for production of silymarin in Ag^+^ elicited *Silybum marianum* hairy root cultures. *J. Med. Plants By Prod.* 1 35–40.

[B35] Kleczkowski K. Schell J. Bandur R. (1995). Phytohormone conjugates, nature and function. *Crit. Rev. Plant Sci.* 14 283–298.

[B36] Küpper H. Lombi E. Zhao F. J. McGrath S. P. (2000). Cellular compartmentation of cadmium and zinc in relation to other elements in the hyperaccumulator *Arabidopsis halleri*. *Planta* 212 75–84. 10.1007/s004250000366 11219586

[B37] Li R. Q. Yu C. Li Y. R. Lam T. W. Yiu S. M. Kristiansen K. (2009). SOAP2, an improved ultrafast tool for short read alignment. *Bioinformatics* 25 1966–1967. 10.1093/bioinformatics/btp336 19497933

[B38] Liu J. P. Zhang C. C. Wei C. C. Liu X. Wang M. G. Yu F. F. (2016). The RING finger ubiquitin E3 ligase OsHTAS enhances heat tolerance by promoting H_2_O_2_-induced stomatal closure in rice. *Plant Physiol.* 170 429–443. 10.1104/pp.15.00879 26564152 PMC4704569

[B39] Liu T. M. Zhu S. Y. Tang Q. M. Tang S. W. (2015). Genome-wide transcriptomic profiling of ramie (*Boehmeria nivea* L. *Gene* 558 131–137. 10.1016/j.gene.2014.12.057 25550046

[B40] Livak K. J. Schmittgen T. D. (2001). Analysis of relative gene expression data using real-time quantitative PCR and the 2^–Δ^ ^Δ^ *^CT^* method. *Methods* 25 402–408. 10.1006/meth.2001.1262 11846609

[B41] Luo J. S. Yang Y. Gu T. Y. Wu Z. M. Zhang Z. H. (2019). The *Arabidopsis* defensin gene AtPDF2.5 mediates cadmium tolerance and accumulation. *Plant Cell Environ.* 42 2681–2695. 10.1111/pce.13592 31115921

[B42] Martin F. Bovet L. Cordier A. Stanke M. Gunduz I. Peitsch M. C. (2012). Design of a tobacco exon array with application to investigate the differential cadmium accumulation property in two tobacco varieties. *BMC Genomics* 13:674–690. 10.1186/1471-2164-13-674 23190529 PMC3602038

[B43] Mazel A. Levine L. (2002). Induction of glucosyltransferase transcription and activity during superoxide-dependent cell death in *Arabidopsis* plants. *Plant Physiol. Biochem.* 40 133–140. 10.1016/s0981-9428(01)01351-1

[B44] Mittler R. (2002). Oxidative stress, antioxidants and stress tolerance. *Trends Plant Sci.* 7 405–410. 10.1016/s1360-1385(02)02312-9 12234732

[B45] Moller I. M. (2001). Plant mitochondria and oxidative stredd: electron transport, NADPH turnover, and metabolism of reactive oxygen species. *Annu. Rev. Plant Physiol. Plant Mol. Biol.* 52 561–591. 10.1146/annurev.arplant.52.1.561 11337409

[B46] Milner M. J. Mitani-Ueno N. Yamaji N. Yokosho K. Craft E. Fei Z. J. (2014). Root and shoot transcriptome analysis of two ecotypes of *Noccaea caerulescens* uncovers the role of NcNramp1 in Cd hyperaccumlation. *Plant J.* 78 398–410. 10.1111/tpj.12480 24547775

[B47] Oono Y. Yazawa T. Kawahara Y. Kanamori H. Kobayashi F. Sasaki H. (2014). Genome-wide transcriptome analysis reveals that cadmium stress signaling controls the expression of genes in drought stress signal pathways in rice. *PLoS One* 9:e96946. 10.1371/journal.pone.0096946 24816929 PMC4016200

[B48] Priest D. M. Ambrose S. J. Vaistij F. E. Elias L. Higgins G. S. Ross A. R. S. (2006). Use of the glucosyltransferase UGT71B6 to disturb abscisic acid homeostasis in *Arabidopsis thaliana*. *Plant J.* 46 492–502. 10.1111/j.1365-313X.2006.02701.x 16623908

[B49] Qin L. Wang L. Guo Y. Li Y. Ümüt H. Wang Y. (2017). An ERF transcription factor from *Tamarix hispida*. *Plant Sci.* 265 154–166. 10.1016/j.plantsci.2017.10.006 29223337

[B50] Rajkumar A. P. Qvist P. Lazarus R. Lescai F. Ju J. Nyegaard M. (2015). Experimental validation of methods for differential gene expression analysis and sample pooling in RNA-seq. *BMC Genomics* 16:548–556. 10.1186/s12864-015-1767-y 26208977 PMC4515013

[B51] Rodriguez-Serrano M. Romero-Puertas M. C. Pazmino D. M. Testillano P. S. Risueno M. C. Del Río L. A. (2009). Cellular response of pea plants to cadmium toxicity, cross talk between reactive oxygen species, nitric oxide, and calcium. *Plant Physiol.* 150 229–243. 10.1104/pp.108.131524 19279198 PMC2675729

[B52] Salt D. E. Wagner G. J. (1993). Cadmium transport across tonoplast of vesicles from oat roots. evidence for a Cd^2+^/H^+^ antiport activity. *J. Biol. Chem.* 268 12297–12302. 10.1016/s0021-9258(18)31388-7 8509367

[B53] Saxena I. Srikanth S. Chen Z. (2016). Cross talk between H_2_O_2_ and interacting signal molecules under plant stress response. *Front. Plant Sci.* 7:570. 10.3389/fpls.2016.00570 27200043 PMC4848386

[B54] Sepulveda-Jimenez G. Rueda-Benıtez P. Porta H. Rocha-Sosa M. (2004). A red beet (*Beta vulgaris*) UDP-glucosyltransferase gene induced by wounding, bacterial infiltration and oxidative stress. *J. Eep. Bot.* 56 605–611. 10.1093/jxb/eri036 15582929

[B55] Sharma S. S. Kumar V. (2002). Responses of wild type and abscisic acid mutants of *Arabidopsis thaliana* to cadmium. *J. Plant Physiol.* 159 1323–1327. 10.1078/0176-1617-00601

[B56] Suwazono Y. Kido T. Nakagawa H. Nishijo M. Nogawa K. (2009). Biological half-life of cadmium in the urine of inhabitants after cessation of cadmium exposure. *Biomarkers* 14 77–81. 10.1080/13547500902730698 19330585

[B57] Tognetti V. B. Van Aken O. Morreel K. Vandenbroucke K. Van de Cotte B. De Clercq I. (2010). Perturbation of indole-3-butyric acid homeostasis by the UDP- glucosyltransferase UGT74E2 modulates *Arabidopsis* architecture and water stress tolerance. *Plant Cell* 22 2660–2679. 10.1105/tpc.109.071316 20798329 PMC2947170

[B58] Uraguchi S. Mori S. Kuramata M. Kawasaki A. Arao T. Ishikawa S. (2009). Root-to-shoot Cd translocation *via* the xylem is the major process determining shoot and grain cadmium accumulation in rice. *J. Eep. Bot.* 60 2677–2688. 10.1093/jxb/erp119 19401409 PMC2692013

[B59] Vandesompele J. De Preter K. Pattyn F. Poppe B. Roy N. V. Paepe A. D. (2002). Accurate normalization of real-time quantitative RT-PCR data by geometric averaging of multiple internal control genes. *Genome Biol. Res.* 3 1–12. 10.1186/gb-2002-3-7-research0034 12184808 PMC126239

[B60] Vogel-Mikus K. Pongrac P. Kump P. Necemer M. Regvar M. (2005). Colonisation of a Zn, Cd and Pb hyperaccumulator *Thlaspi praecox* wulfen with indigenous arbuscular mycorrhizal fungal mixture induces changes in heavy metal and nutrient uptake. *Environ. Pollut.* 139 362–371. 10.1016/j.envpol.2005.05.005 15998561

[B61] Wan L. C. Zhang H. Y. (2012). Cadmium toxicity, effects on cytoskeleton, vesicular trafficking and cell wall construction. *Plant Signal. Behav.* 7 345–348. 10.4161/psb.18992 22499203 PMC3443916

[B62] Wang C. Gao C. Wang L. Zheng L. Yang C. Wang Y. (2013). Comprehensive transcriptional profiling of NaHCO3-stressed *Tamarix hispida* roots reveals networks of responsive genes. *Plant Mol. Biol.* 84 145–157. 10.1007/s11103-013-0124-2 24022749

[B63] Wang P. L. Lei X. Lü J. X. Gao C. Q. (2020). Overexpression of the ThTPS gene enhanced salt and osmotic stress tolerance in *Tamarix hispida*. *J. For. Res.* 1–10.

[B64] Wei S. H. Zhou Q. X. Wang X. (2005). Cadmium Hyperaccumulator *Solanum nigrum* L. and its accumulating characteristics. *Environ. sci.* 26 167–171. 16124492

[B65] Wu F. B. Zhang G. P. Dominy P. Wu H. X. Bachir Dango M. L. (2007). Differences in yield components and kernel Cd accumulation in response to Cd toxicity in four barley genotypes. *Chemosphere* 70 83–92. 10.1016/j.chemosphere.2007.06.051 17675207

[B66] Xu S. S. Lin S. Z. Lai Z. X. (2015). Cadmium impairs iron homeostasis in Arabidopsis thaliana by increasing the polysaccharide contents and the iron-binding capacity of root cell walls. *Plant Soil* 392 71–85. 10.1007/s11104-015-2443-3

[B67] Xu Z. J. Nakajima M. Suzuki Y. Yamaguchi I. (2002). Cloning and characterization of the abscisic acid-specific glucosyltransferase gene from Adzuki bean seedings. *J. Plant Physiol.* 129 1285–1295. 10.1104/pp.001784 12114582 PMC166522

[B68] Yang G. Y. Wang C. Wang Y. C. Guo Y. C. Zhao Y. L. Yang C. P. (2016). Overexpression of ThVHAc1 and its potential upstream regulator, ThWRKY7, improved plant tolerance of cadmium stress. *Sci. Rep.* 6:18752. 10.1038/srep18752 26744182 PMC4705465

[B69] Yang J. L. Wang Y. C. Liu G. F. Yang C. P. Li C. H. (2011). Tamarix hispida metallothionein-like ThMT3, a reactive oxygen species scavenger, increases tolerance against Cd2+, Zn2+, Cu2+, and NaCl in transgenic yeast. *Mol Biol Rep.* 38 1567–1574. 10.1007/s11033-010-0265-1 20835888

[B70] Yang X. E. Long X. X. Ye H. B. He Z. L. Calver D. V. Stofflla P. J. (2004). Cadmium tolerance and hyperaccumulation in a new Zn-hyperaccumulating plant species (*Sedum alfredii Hance*). *Plant Soil.* 259 181–189. 10.1023/b:plso.0000020956.24027.f2

[B71] Ye J. Fang L. Zheng H. K. Zhang Y. Chen J. Zhang Z. J. (2006). WEGO, a web tool for plotting go annotations. *Nucleic Acids Res.* 34 293–297. 10.1093/nar/gkl031 PMC1538768 16845012

[B72] Yue R. Q. Lu C. X. Qi J. S. Han X. H. Yan S. F. Guo S. L. (2016). Transcriptome analysis of cadmium-treated roots in maize (*Zea mays* L.). *Front. Plant Sci.* 7:1298. 10.3389/fpls.2016.01298 27630647 PMC5006096

[B73] Zang D. Wang C. Ji X. Wang Y. (2015). *Tamarix hispida* zinc finger protein ThZFP1 participates in salt and osmotic stress tolerance by increasing proline content and SOD and POD activities. *Plant Sci.* 235 111–121. 10.1016/j.plantsci.2015.02.016 25900571

[B74] Zhang Z. H. Zhou T. Tang T. J. Song H. Q. Guan C. Y. Huang J. S. (2019b). Multiomics landscapes uncover the pivotal role of subcellular reallocation of cadmium in regulating rapeseed resistance to cadmium toxicity. *J. Exp. Bot.* 2019:erz295. 10.1093/jxb/erz295 PMC6793439 31232451

[B75] Zhang P. Wang R. L. Ju Q. Li W. Q. Tran L. P. Jin X. (2019a). The R2R3-MYB transcription factor MYB49 regulates cadmium accumulation. *Plant Physiol.* 180 529–542. 10.1104/pp.18.01380 30782964 PMC6501104

[B76] Zhang X. Wang L. Meng H. Wen H. Fan Y. Zhao J. (2011). Maize ABP9 enhances tolerance to multiple stresses in transgenic *Arabidopsis* by modulating ABA signaling and cellular levels of reactive oxygen species. *Plant Mol. Biol.* 75 365–378. 10.1007/s11103-011-9732-x 21327835 PMC3044229

[B77] Zhou B. R. Yao W. J. Wang S. J. Wang X. W. Jiang T. B. (2014). The metallothionein gene, tamt3, from tamarix androssowii confers cd2+ tolerance in tobacco. *Int. J. Mol. Sci.* 15 10398–10409. 10.3390/ijms150610398 24918294 PMC4100158

[B78] Zhou Q. Guo J. J. He C. S. Shen C. Huang Y. Y. Chen J. X. (2016). Comparative transcriptome analysis between low- and high- cadmium -accumulating genotypes of pakchoi (*Brassica chinensis* L.) in response to cadmium stress. *Environ. Sci. Technol.* 50 6485–6494. 10.1021/acs.est.5b06326 27228483

[B79] Zuccarelli R. Coelho A. C. P. Peres L. E. P. Freschi L. (2017). Shedding light on no homeostasis, light as a key regulator of glutathione and nitric oxide metabolisms during seedling deetiolation. *Nitric Oxide* 68 77–90. 10.1016/j.niox.2017.01.006 28109803

