# Supplementary material for: Transcriptomic Analysis of Cadmium Stressed Tamarix hispida Revealed Novel Transcripts and the Importance of Abscisic Acid Network
Source: Front Plant Sci. 2022 Apr 18;13:843725. doi: 10.3389/fpls.2022.843725 (PMC9062237; doi:10.3389/fpls.2022.843725)
Supplement: Supplementary file 1 [file Table_1.DOCX]

**Supplementary Table 1**

|  | 24 h vs control | 48 h vs control | 72 h vs control |
| --- | --- | --- | --- |
| Total gene numbers | 6778 | 8282 | 8601 |
| Genes have similar sequences in the NR database | 4105 | 5224 | 5524 |
| Genes have no similar sequences in the NR database | 2673 | 3058 | 3077 |

**DEGs numbers about similar sequences in the NR database**
