# Supplementary material for: Transcriptomic Analysis of Cadmium Stressed Tamarix hispida Revealed Novel Transcripts and the Importance of Abscisic Acid Network
Source: Front Plant Sci. 2022 Apr 18;13:843725. doi: 10.3389/fpls.2022.843725 (PMC9062237; doi:10.3389/fpls.2022.843725)
Supplement: Supplementary file 2 [file Table_2.DOCX]

| **Supplementary Table 2** | | | |
| --- | --- | --- | --- |
| **Gene IDs, descriptions and primer sequences of the eight candidate genes and internal control used for qRT-PCR** | | | |
| Gene ID | Gene description | Primer | Sequence (5’ → 3’) |
| XP_010272925.1 | aspartic proteinase nepenthesin-1-like | asp-F | ATGCTCGGGGTGTACCGGTT |
|  |  | asp-R | GCATTAATGCATCCGAAGAC |
| XP_008241363.1 | UDP-glycosyltransferase 73C1-like | UDP-F | TGCGTAAGACCAAGGTGCAT |
|  |  | UDP-R | CCTCTTTGCCTTATCCGAACT |
| XP_007051612.1 | Indole-3-acetic acid-amido synthetase GH3.5, putative isoform 1 | Indole-F | GTAAGACAGAAACAGGCAT |
|  |  | Indole-R | ATAGCTGCGCGTAGAGACG |
| XP_008459234.1 | cell division cycle-associated 7-like protein | CELL-F | AGACAATCCCCAGAAACAAGAC |
|  |  | CELL-R | TCGACCCTTCAGGAATATG |
| XP_012440061.1 | hevamine-A-like | hevamine-F | AGCGTAGGTAGCGATGTAAAG |
|  |  | hevamine-R | ATCGCTCCTTGCAGGTGGGC |
| XP_009776344.1 | protein ZINC INDUCED FACILITATOR-LIKE 1-like isoform X1 | ZFL-F | AGGACCAGTGAAGGCTTAT |
|  |  | ZFL-R | AGTGACGTGCATAGGCAAGG |
| XP_010657270.1 | probable protein phosphatase 2C 52 | PP2C-F | ATACTGACCAGGTTGATTCC |
|  |  | PP2C-R | AAGAGTCATTCCTGTCCTT |
| XP_009360333.1 | extracellular ribonuclease LE-like | ELE-F | ACGGGTCGTACCCTTCCAACT |
|  |  | ELE-R | ACTCTGTCCGTTCGGCTCGAT |
| FJ618519 | β-actin | actin-F | AAACAATGGCTGATGCTG |
|  |  | actin-R | ACAATACCGTGCTCAATAGG |
