# Supplementary material for: Transcriptomic Analysis of Cadmium Stressed Tamarix hispida Revealed Novel Transcripts and the Importance of Abscisic Acid Network
Source: Front Plant Sci. 2022 Apr 18;13:843725. doi: 10.3389/fpls.2022.843725 (PMC9062237; doi:10.3389/fpls.2022.843725)
Supplement: Supplementary file 3 [file Table_3.DOCX]

**Supplementary Table 3**

**24 h DEGs function annotation and gene number statistics.**

| Description | DEG_item |
| --- | --- |
| tRNA binding | 1 |
| phosphorelay sensor kinase activity | 3 |
| nucleotide binding | 104 |
| DNA secondary structure binding | 1 |
| magnesium ion binding | 3 |
| 3-hydroxyanthranilate 3,4-dioxygenase activity | 1 |
| four-way junction DNA binding | 1 |
| transcription factor activity, protein binding | 6 |
| transcription factor activity, transcription factor binding | 5 |
| transcription factor activity, core RNA polymerase binding | 1 |
| core DNA-dependent RNA polymerase binding promoter specificity activity | 1 |
| nucleic acid binding transcription factor activity | 32 |
| transcription factor activity, RNA polymerase II transcription factor binding | 4 |
| RNA polymerase II transcription cofactor activity | 4 |
| G-protein coupled receptor binding | 1 |
| pattern binding | 3 |
| nucleoside binding | 85 |
| purine nucleoside binding | 85 |
| nucleic acid binding | 107 |
| DNA binding | 74 |
| DNA helicase activity | 6 |
| chromatin binding | 1 |
| double-stranded DNA binding | 2 |
| single-stranded DNA binding | 2 |
| transcription factor activity, sequence-specific DNA binding | 32 |
| transcription cofactor activity | 5 |
| transcription corepressor activity | 1 |
| RNA binding | 23 |
| RNA helicase activity | 4 |
| structural constituent of ribosome | 3 |
| translation initiation factor activity | 1 |
| peptidyl-prolyl cis-trans isomerase activity | 1 |
| motor activity | 2 |
| microtubule motor activity | 1 |
| actin binding | 1 |
| catalytic activity | 393 |
| 3-deoxy-7-phosphoheptulonate synthase activity | 1 |
| 3-beta-hydroxy-delta5-steroid dehydrogenase activity | 1 |
| 5-aminolevulinate synthase activity | 1 |
| 5-methyltetrahydropteroyltriglutamate-homocysteine S-methyltransferase activity | 1 |
| DNA-directed DNA polymerase activity | 4 |
| DNA primase activity | 1 |
| DNA-directed RNA polymerase activity | 6 |
| DNA ligase activity | 3 |
| DNA ligase (ATP) activity | 1 |
| DNA ligase (NAD+) activity | 2 |
| DNA topoisomerase activity | 1 |
| FMN adenylyltransferase activity | 1 |
| GTPase activity | 12 |
| NADH dehydrogenase activity | 2 |
| RNA-directed RNA polymerase activity | 2 |
| acid phosphatase activity | 5 |
| cytidine deaminase activity | 1 |
| cytidylate kinase activity | 3 |
| dimethylallyltranstransferase activity | 1 |
| endopeptidase activity | 19 |
| ATP-dependent peptidase activity | 2 |
| aminopeptidase activity | 3 |
| carboxypeptidase activity | 11 |
| serine-type carboxypeptidase activity | 11 |
| aspartic-type endopeptidase activity | 6 |
| cysteine-type endopeptidase activity | 2 |
| metalloendopeptidase activity | 2 |
| serine-type endopeptidase activity | 10 |
| fructose-bisphosphate aldolase activity | 2 |
| glucose-6-phosphate dehydrogenase activity | 1 |
| glucosylceramidase activity | 3 |
| glutamate-ammonia ligase activity | 1 |
| glutamate-cysteine ligase activity | 1 |
| glutamate N-acetyltransferase activity | 1 |
| glutathione synthase activity | 1 |
| helicase activity | 6 |
| hydroxymethylglutaryl-CoA reductase (NADPH) activity | 1 |
| indole-3-glycerol-phosphate synthase activity | 1 |
| malic enzyme activity | 1 |
| malate dehydrogenase (decarboxylating) (NAD+) activity | 1 |
| methionine adenosyltransferase activity | 3 |
| methylenetetrahydrofolate reductase (NAD(P)H) activity | 1 |
| monooxygenase activity | 3 |
| nuclease activity | 15 |
| endonuclease activity | 14 |
| endodeoxyribonuclease activity | 4 |
| endoribonuclease activity | 7 |
| ribonuclease III activity | 4 |
| exonuclease activity | 1 |
| exodeoxyribonuclease activity | 1 |
| deoxyribonuclease activity | 5 |
| ribonuclease activity | 7 |
| hydrolase activity, hydrolyzing O-glycosyl compounds | 44 |
| beta-galactosidase activity | 1 |
| pantoate-beta-alanine ligase activity | 1 |
| peroxidase activity | 10 |
| glutathione peroxidase activity | 1 |
| phosphatidylserine decarboxylase activity | 1 |
| phosphogluconate dehydrogenase (decarboxylating) activity | 1 |
| phospholipase activity | 1 |
| phospholipase A2 activity | 1 |
| polygalacturonase activity | 2 |
| porphobilinogen synthase activity | 1 |
| prenyltransferase activity | 1 |
| prephenate dehydratase activity | 1 |
| protein kinase activity | 48 |
| protein histidine kinase activity | 4 |
| protein serine/threonine kinase activity | 9 |
| DNA-dependent protein kinase activity | 1 |
| calmodulin-dependent protein kinase activity | 1 |
| protein tyrosine kinase activity | 2 |
| non-membrane spanning protein tyrosine kinase activity | 1 |
| ribose-5-phosphate isomerase activity | 1 |
| transposase activity | 1 |
| triose-phosphate isomerase activity | 1 |
| tRNA (guanine-N2-)-methyltransferase activity | 1 |
| aminoacyl-tRNA ligase activity | 2 |
| alanine-tRNA ligase activity | 1 |
| ubiquitin-protein transferase activity | 13 |
| enzyme inhibitor activity | 3 |
| protein kinase inhibitor activity | 1 |
| cyclin-dependent protein serine/threonine kinase inhibitor activity | 1 |
| signal transducer activity | 7 |
| receptor activity | 6 |
| transmembrane signaling receptor activity | 3 |
| G-protein coupled receptor activity | 1 |
| ionotropic glutamate receptor activity | 1 |
| receptor signaling protein activity | 3 |
| guanyl-nucleotide exchange factor activity | 1 |
| ARF guanyl-nucleotide exchange factor activity | 1 |
| GTPase activator activity | 3 |
| receptor binding | 11 |
| cytokine activity | 1 |
| cytokine receptor binding | 6 |
| interferon-gamma receptor binding | 1 |
| interleukin-4 receptor binding | 1 |
| interleukin-1 receptor binding | 1 |
| stem cell factor receptor binding | 2 |
| hormone activity | 2 |
| structural molecule activity | 20 |
| structural constituent of cell wall | 2 |
| structural constituent of eye lens | 2 |
| structural constituent of chorion | 1 |
| transporter activity | 66 |
| ion channel activity | 8 |
| extracellular ligand-gated ion channel activity | 1 |
| voltage-gated ion channel activity | 1 |
| calcium channel regulator activity | 1 |
| voltage-gated potassium channel activity | 1 |
| cation channel activity | 3 |
| potassium channel activity | 1 |
| inorganic phosphate transmembrane transporter activity | 2 |
| copper ion transmembrane transporter activity | 1 |
| iron ion transmembrane transporter activity | 1 |
| binding | 385 |
| iron ion binding | 20 |
| copper ion binding | 8 |
| calcium ion binding | 17 |
| protein binding | 175 |
| calmodulin binding | 3 |
| ATP binding | 77 |
| GTP binding | 14 |
| phospholipid binding | 4 |
| calcium-dependent phospholipid binding | 3 |
| chemokine activity | 1 |
| microtubule binding | 2 |
| enzyme activator activity | 3 |
| chitin binding | 1 |
| glutamate receptor activity | 1 |
| N-acetyltransferase activity | 1 |
| growth factor activity | 2 |
| cytoskeletal protein binding | 8 |
| ubiquinol-cytochrome-c reductase activity | 1 |
| transcription factor binding | 10 |
| translation factor activity, RNA binding | 1 |
| NADH dehydrogenase (ubiquinone) activity | 2 |
| sulfotransferase activity | 8 |
| hedgehog receptor activity | 2 |
| methyltransferase activity | 12 |
| N-methyltransferase activity | 1 |
| O-methyltransferase activity | 2 |
| S-methyltransferase activity | 1 |
| RNA methyltransferase activity | 2 |
| tRNA methyltransferase activity | 1 |
| UDP-glycosyltransferase activity | 6 |
| ferric iron binding | 1 |
| ion channel inhibitor activity | 3 |
| peptidase activity | 38 |
| cysteine-type peptidase activity | 3 |
| serine-type peptidase activity | 23 |
| metallopeptidase activity | 2 |
| exopeptidase activity | 16 |
| dipeptidyl-peptidase activity | 1 |
| zinc ion binding | 41 |
| lipid binding | 8 |
| cation transmembrane transporter activity | 21 |
| O-acyltransferase activity | 2 |
| galactosyltransferase activity | 1 |
| selenium binding | 1 |
| glycogenin glucosyltransferase activity | 1 |
| anion transmembrane transporter activity | 2 |
| ammonium transmembrane transporter activity | 1 |
| Ran GTPase binding | 3 |
| protein transporter activity | 1 |
| 1-deoxy-D-xylulose-5-phosphate synthase activity | 2 |
| D-alanine-D-alanine ligase activity | 1 |
| S-adenosylmethionine-dependent methyltransferase activity | 1 |
| dCTP deaminase activity | 1 |
| 4-hydroxy-tetrahydrodipicolinate reductase | 1 |
| exodeoxyribonuclease VII activity | 1 |
| lipopolysaccharide 3-alpha-galactosyltransferase activity | 1 |
| oxaloacetate decarboxylase activity | 1 |
| phospho-N-acetylmuramoyl-pentapeptide-transferase activity | 1 |
| quinolinate synthetase A activity | 1 |
| starch synthase activity | 2 |
| Type II site-specific deoxyribonuclease activity | 4 |
| electron carrier activity | 8 |
| four-way junction helicase activity | 5 |
| plastoquinol--plastocyanin reductase activity | 1 |
| pseudouridine synthase activity | 1 |
| FMN binding | 1 |
| acireductone dioxygenase [iron(II)-requiring] activity | 1 |
| terpene synthase activity | 1 |
| sesquiterpene synthase activity | 1 |
| protein disulfide oxidoreductase activity | 2 |
| disulfide oxidoreductase activity | 2 |
| ion transmembrane transporter activity | 28 |
| monovalent inorganic cation transmembrane transporter activity | 10 |
| hydrogen ion transmembrane transporter activity | 7 |
| potassium ion transmembrane transporter activity | 1 |
| sodium ion transmembrane transporter activity | 1 |
| ferrous iron transmembrane transporter activity | 1 |
| magnesium ion transmembrane transporter activity | 1 |
| mercury ion transmembrane transporter activity | 2 |
| molybdate ion transmembrane transporter activity | 1 |
| inorganic anion transmembrane transporter activity | 2 |
| phosphate ion transmembrane transporter activity | 1 |
| carbohydrate transmembrane transporter activity | 1 |
| drug transmembrane transporter activity | 4 |
| channel activity | 8 |
| ligand-gated ion channel activity | 1 |
| secondary active transmembrane transporter activity | 7 |
| antiporter activity | 5 |
| solute:cation antiporter activity | 1 |
| solute:proton antiporter activity | 1 |
| primary active transmembrane transporter activity | 5 |
| P-P-bond-hydrolysis-driven transmembrane transporter activity | 5 |
| tubulin binding | 4 |
| restriction endodeoxyribonuclease activity | 4 |
| galactosidase activity | 1 |
| glutamate synthase activity | 1 |
| nickel cation binding | 2 |
| antioxidant activity | 13 |
| ammonia ligase activity | 1 |
| steroid dehydrogenase activity | 1 |
| channel regulator activity | 4 |
| channel inhibitor activity | 3 |
| lipase activity | 1 |
| kinase activity | 56 |
| phosphatidylinositol phosphate kinase activity | 1 |
| acetyltransferase activity | 1 |
| N-acyltransferase activity | 2 |
| tRNA (guanine) methyltransferase activity | 1 |
| pyrophosphatase activity | 23 |
| oxidoreductase activity | 87 |
| cyclin-dependent protein serine/threonine kinase regulator activity | 1 |
| amino acid binding | 4 |
| oxidoreductase activity, acting on CH-OH group of donors | 8 |
| malate dehydrogenase activity | 1 |
| oxidoreductase activity, acting on the CH-OH group of donors, NAD or NADP as acceptor | 6 |
| oxidoreductase activity, acting on the aldehyde or oxo group of donors, disulfide as acceptor | 2 |
| oxidoreductase activity, acting on the CH-CH group of donors | 4 |
| oxidoreductase activity, acting on the CH-CH group of donors, NAD or NADP as acceptor | 2 |
| oxidoreductase activity, acting on the CH-NH2 group of donors | 1 |
| oxidoreductase activity, acting on the CH-NH group of donors | 1 |
| oxidoreductase activity, acting on the CH-NH group of donors, NAD or NADP as acceptor | 1 |
| oxidoreductase activity, acting on NAD(P)H | 8 |
| oxidoreductase activity, acting on NAD(P)H, quinone or similar compound as acceptor | 3 |
| oxidoreductase activity, acting on a sulfur group of donors | 6 |
| oxidoreductase activity, acting on a sulfur group of donors, NAD(P) as acceptor | 4 |
| oxidoreductase activity, acting on diphenols and related substances as donors | 2 |
| oxidoreductase activity, acting on diphenols and related substances as donors, cytochrome as acceptor | 1 |
| oxidoreductase activity, acting on peroxide as acceptor | 10 |
| L-ascorbate peroxidase activity | 1 |
| oxidoreductase activity, acting on single donors with incorporation of molecular oxygen | 8 |
| oxidoreductase activity, acting on single donors with incorporation of molecular oxygen, incorporation of two atoms of oxygen | 2 |
| oxidoreductase activity, acting on single donors with incorporation of molecular oxygen, incorporation of one atom of oxygen (internal monooxygenases or internal mixed function oxidases) | 2 |
| oxidoreductase activity, acting on paired donors, with incorporation or reduction of molecular oxygen | 11 |
| oxidoreductase activity, acting on paired donors, with incorporation or reduction of molecular oxygen, 2-oxoglutarate as one donor, and incorporation of one atom each of oxygen into both donors | 3 |
| oxidoreductase activity, acting on paired donors, with incorporation or reduction of molecular oxygen, NAD(P)H as one donor, and incorporation of one atom of oxygen | 1 |
| oxidoreductase activity, acting on paired donors, with oxidation of a pair of donors resulting in the reduction of molecular oxygen to two molecules of water | 1 |
| oxidoreductase activity, acting on CH or CH2 groups | 1 |
| oxidoreductase activity, acting on CH or CH2 groups, NAD or NADP as acceptor | 1 |
| transferase activity | 161 |
| transferase activity, transferring one-carbon groups | 12 |
| transferase activity, transferring aldehyde or ketonic groups | 2 |
| transferase activity, transferring acyl groups | 12 |
| transferase activity, transferring acyl groups other than amino-acyl groups | 10 |
| succinyltransferase activity | 1 |
| N-succinyltransferase activity | 1 |
| transferase activity, transferring glycosyl groups | 28 |
| transferase activity, transferring hexosyl groups | 23 |
| cellulose synthase activity | 4 |
| cellulose synthase (UDP-forming) activity | 4 |
| xyloglucan:xyloglucosyl transferase activity | 5 |
| transferase activity, transferring alkyl or aryl (other than methyl) groups | 9 |
| transferase activity, transferring phosphorus-containing groups | 80 |
| phosphotransferase activity, alcohol group as acceptor | 55 |
| phosphotransferase activity, nitrogenous group as acceptor | 4 |
| nucleotidyltransferase activity | 15 |
| phosphotransferase activity, for other substituted phosphate groups | 2 |
| transferase activity, transferring sulfur-containing groups | 9 |
| hydrolase activity | 149 |
| hydrolase activity, acting on ester bonds | 44 |
| phosphatase activity | 8 |
| exonuclease activity, active with either ribo- or deoxyribonucleic acids and producing 5'-phosphomonoesters | 1 |
| hydrolase activity, acting on glycosyl bonds | 48 |
| hydrolase activity, hydrolyzing N-glycosyl compounds | 1 |
| hydrolase activity, acting on carbon-nitrogen (but not peptide) bonds | 2 |
| hydrolase activity, acting on carbon-nitrogen (but not peptide) bonds, in cyclic amidines | 2 |
| hydrolase activity, acting on acid anhydrides | 29 |
| hydrolase activity, acting on acid anhydrides, in phosphorus-containing anhydrides | 27 |
| hydrolase activity, acting on acid anhydrides, catalyzing transmembrane movement of substances | 6 |
| lyase activity | 18 |
| carbon-carbon lyase activity | 7 |
| carboxy-lyase activity | 5 |
| aldehyde-lyase activity | 2 |
| carbon-oxygen lyase activity | 4 |
| hydro-lyase activity | 3 |
| carbon-oxygen lyase activity, acting on phosphates | 1 |
| carbon-nitrogen lyase activity | 2 |
| ammonia-lyase activity | 1 |
| amidine-lyase activity | 1 |
| carbon-sulfur lyase activity | 2 |
| isomerase activity | 11 |
| cis-trans isomerase activity | 1 |
| intramolecular oxidoreductase activity | 2 |
| intramolecular oxidoreductase activity, interconverting aldoses and ketoses | 2 |
| intramolecular transferase activity | 3 |
| ligase activity | 11 |
| ligase activity, forming carbon-oxygen bonds | 3 |
| ligase activity, forming aminoacyl-tRNA and related compounds | 3 |
| ligase activity, forming carbon-nitrogen bonds | 5 |
| acid-ammonia (or amide) ligase activity | 1 |
| acid-amino acid ligase activity | 4 |
| ligase activity, forming phosphoric ester bonds | 3 |
| ATPase activity | 13 |
| endodeoxyribonuclease activity, producing 5'-phosphomonoesters | 4 |
| endoribonuclease activity, producing 5'-phosphomonoesters | 4 |
| endoribonuclease activity, producing 3'-phosphomonoesters | 3 |
| endonuclease activity, active with either ribo- or deoxyribonucleic acids and producing 5'-phosphomonoesters | 8 |
| endonuclease activity, active with either ribo- or deoxyribonucleic acids and producing 3'-phosphomonoesters | 3 |
| exodeoxyribonuclease activity, producing 5'-phosphomonoesters | 1 |
| oxidoreductase activity, acting on the aldehyde or oxo group of donors | 2 |
| sigma factor activity | 1 |
| Ras GTPase binding | 3 |
| myosin binding | 2 |
| purine nucleotide binding | 85 |
| sodium channel regulator activity | 1 |
| nucleoside-triphosphatase activity | 23 |
| tRNA dihydrouridine synthase activity | 2 |
| serine hydrolase activity | 23 |
| nitronate monooxygenase activity | 2 |
| guanyl nucleotide binding | 14 |
| nucleotide kinase activity | 3 |
| nucleobase-containing compound kinase activity | 3 |
| kinase regulator activity | 1 |
| kinase inhibitor activity | 1 |
| deaminase activity | 2 |
| ubiquitin-like protein transferase activity | 13 |
| oxygen binding | 4 |
| cation-transporting ATPase activity | 1 |
| vitamin binding | 3 |
| sodium channel inhibitor activity | 1 |
| protein kinase regulator activity | 1 |
| enzyme binding | 8 |
| kinase binding | 5 |
| protein kinase binding | 5 |
| heme binding | 23 |
| passive transmembrane transporter activity | 8 |
| active transmembrane transporter activity | 12 |
| voltage-gated channel activity | 1 |
| ligand-gated channel activity | 1 |
| gated channel activity | 2 |
| substrate-specific channel activity | 8 |
| voltage-gated cation channel activity | 1 |
| transmembrane transporter activity | 53 |
| inorganic cation transmembrane transporter activity | 18 |
| substrate-specific transmembrane transporter activity | 29 |
| substrate-specific transporter activity | 30 |
| manganese ion binding | 3 |
| pyridoxal phosphate binding | 8 |
| enzyme regulator activity | 8 |
| carbohydrate binding | 5 |
| polysaccharide binding | 3 |
| cellulose binding | 1 |
| protein serine/threonine kinase inhibitor activity | 1 |
| adenyl nucleotide binding | 77 |
| RNA glycosylase activity | 1 |
| rRNA N-glycosylase activity | 1 |
| pectinesterase activity | 2 |
| protein binding, bridging | 2 |
| GTPase regulator activity | 3 |
| potassium ion binding | 1 |
| thiamine pyrophosphate binding | 3 |
| mismatched DNA binding | 1 |
| heat shock protein binding | 2 |
| small GTPase binding | 3 |
| carboxylic acid binding | 4 |
| alkali metal ion binding | 1 |
| nucleosome binding | 1 |
| ubiquitin-like protein binding | 1 |
| double-stranded RNA-specific ribonuclease activity | 4 |
| protein complex binding | 4 |
| ribonucleoside binding | 85 |
| purine ribonucleoside binding | 85 |
| ribonucleotide binding | 86 |
| purine ribonucleotide binding | 85 |
| adenyl ribonucleotide binding | 77 |
| guanyl ribonucleotide binding | 14 |
| acetylcholine receptor binding | 1 |
| steroid dehydrogenase activity, acting on the CH-OH group of donors, NAD or NADP as acceptor | 1 |
| ribonuclease T2 activity | 3 |
| xylan endo-1,3-beta-xylosidase activity | 1 |
| mannosyl-glycoprotein endo-beta-N-acetylglucosaminidase activity | 1 |
| DNA polymerase activity | 4 |
| RNA polymerase activity | 8 |
| UDP-galactosyltransferase activity | 1 |
| UDP-glucosyltransferase activity | 5 |
| purine ribonucleoside triphosphate binding | 85 |
| small molecule binding | 111 |
| hydrogen-exporting ATPase activity | 1 |
| signaling receptor activity | 6 |
| ATPase inhibitor activity | 1 |
| 5-methyltetrahydropteroyltri-L-glutamate-dependent methyltransferase activity | 1 |
| chemokine receptor binding | 1 |
| phosphoric ester hydrolase activity | 10 |
| ATPase activity, coupled | 7 |
| ATPase activity, coupled to transmembrane movement of ions | 1 |
| ATPase activity, coupled to transmembrane movement of substances | 5 |
| identical protein binding | 7 |
| protein homodimerization activity | 6 |
| gamma-tubulin binding | 1 |
| precorrin-2 dehydrogenase activity | 1 |
| ubiquitin binding | 1 |
| ion binding | 210 |
| anion binding | 111 |
| cation binding | 113 |
| organic acid binding | 4 |
| ATPase activity, coupled to movement of substances | 5 |
| protein anchor | 1 |
| ADP binding | 2 |
| sequence-specific DNA binding | 17 |
| structure-specific DNA binding | 5 |
| ATPase activity, coupled to transmembrane movement of ions, rotational mechanism | 1 |
| macromolecular complex binding | 4 |
| translation regulator activity | 1 |
| acyl-[acyl-carrier-protein] desaturase activity | 1 |
| trichodiene synthase activity | 1 |
| dynein binding | 6 |
| phenylalanine ammonia-lyase activity | 1 |
| nutrient reservoir activity | 1 |
| SMAD binding | 1 |
| glucosyltransferase activity | 7 |
| alpha-L-arabinofuranosidase activity | 3 |
| metal ion binding | 111 |
| metal ion transmembrane transporter activity | 10 |
| tetrapyrrole binding | 23 |
| transferase activity, transferring acyl groups, acyl groups converted into alkyl on transfer | 1 |
| transition metal ion binding | 69 |
| transition metal ion transmembrane transporter activity | 4 |
| proton-transporting ATPase activity, rotational mechanism | 1 |
| protein dimerization activity | 18 |
| protein-disulfide reductase activity | 4 |
| chlorophyllase activity | 2 |
| arogenate dehydratase activity | 1 |
| cofactor binding | 26 |
| magnesium-protoporphyrin IX monomethyl ester (oxidative) cyclase activity | 1 |
| inositol oxygenase activity | 4 |
| NADH dehydrogenase (quinone) activity | 2 |
| oleate hydratase activity | 1 |
| ureidoglycolate lyase activity | 1 |
| transferase activity, transferring alkylthio groups | 1 |
| coenzyme-B sulfoethylthiotransferase activity | 1 |
| flavin adenine dinucleotide binding | 6 |
| NADP binding | 2 |
| coenzyme binding | 18 |
| actin filament binding | 1 |
| GTPase binding | 3 |
| unfolded protein binding | 9 |
| chaperone binding | 2 |
| dioxygenase activity | 5 |
| NAD binding | 3 |
| iron-sulfur cluster binding | 6 |
| 2 iron, 2 sulfur cluster binding | 2 |
| 4 iron, 4 sulfur cluster binding | 1 |
| metal cluster binding | 6 |
| 4-hydroxy-3-methylbut-2-en-1-yl diphosphate reductase activity | 1 |
| carboxylic ester hydrolase activity | 5 |
| oxidoreductase activity, acting on diphenols and related substances as donors, with copper protein as acceptor | 1 |
| molecular transducer activity | 7 |
| binding, bridging | 2 |
| nucleoside-triphosphatase regulator activity | 4 |
| ATPase regulator activity | 1 |
| ubiquitin protein ligase activity | 2 |
| ubiquitin-like protein ligase activity | 2 |
| aspartic-type peptidase activity | 6 |
| serine-type exopeptidase activity | 14 |
| serine-type aminopeptidase activity | 1 |
| peptidase activity, acting on L-amino acid peptides | 37 |
| NADPH binding | 1 |
| adenylyltransferase activity | 1 |
| growth factor receptor binding | 2 |
| FAD binding | 1 |
| divalent inorganic cation transmembrane transporter activity | 4 |
| drug transporter activity | 4 |
| organic cyclic compound binding | 216 |
| carbohydrate derivative binding | 87 |
| xylanase activity | 1 |
| nucleotide phosphatase activity, acting on free nucleotides | 3 |
| molecular function regulator | 12 |
| nucleoside phosphate binding | 104 |
| heterocyclic compound binding | 216 |
| carbohydrate transporter activity | 1 |
| phosphate transmembrane transporter activity | 2 |
| sulfur compound binding | 3 |
| starch binding | 1 |
| total | 6601 |

Note: DEGs participated in the redox reaction, the transport of ions, and the synthesis of signal substances have a yellow background.
