# Supplementary figures and images for: Transcriptomic Analysis of Cadmium Stressed Tamarix hispida Revealed Novel Transcripts and the Importance of Abscisic Acid Network
Source: Front Plant Sci. 2022 Apr 18;13:843725. doi: 10.3389/fpls.2022.843725 (PMC9062237; doi:10.3389/fpls.2022.843725)

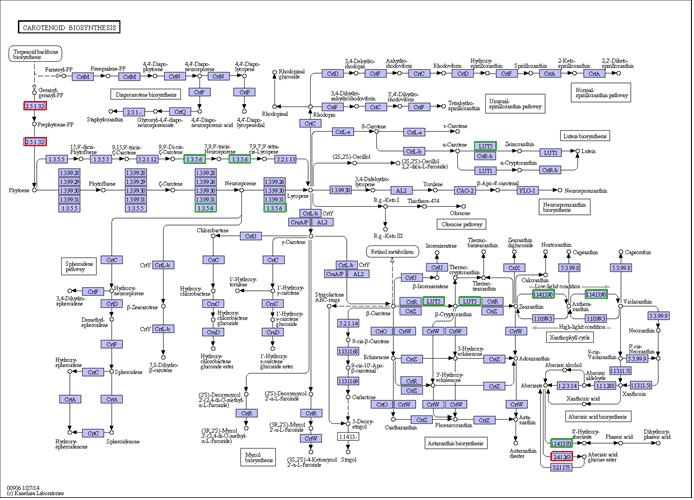

Supplement: Supplementary Figure 1 — Signaling pathways involved in ABA. The genes associated with ABA were marked in different colors. Red: up-regulated genes. Blue: down-regulated genes. [file Image_1.JPEG]
